# Syncope: Diagnostic Yield of Various Clinical Investigations

**DOI:** 10.7759/cureus.23596

**Published:** 2022-03-29

**Authors:** Rajesh Bhat Uppoor, Kashyap Patel

**Affiliations:** 1 Cardiology, Kasturba Medical College and Hospital, Mangaluru, IND; 2 Medicine, Kasturba Medical College and Hospital, Mangaluru, IND

**Keywords:** clinical investigations, syncope, etiopathogenesis, etiology, diagnostic tests

## Abstract

Objective: The present study was designed to evaluate the clinical profile of patients with syncope and the usefulness of various tests to reach a diagnosis of syncope and its etiology.

Methods: This was a cross-sectional, observational study that enrolled 90 consecutive patients (aged ≥ 12 years) who presented with syncope. Detailed information was obtained from each enrolled patient on history and physical examination. All patients underwent electrocardiography (ECG) and echocardiographic examination. Other specific tests were also performed based on the findings from medical history, physical examination, ECG, and echocardiography findings.

Results: Among 90 patients with syncope, 45% were males, and age distribution showed a bimodal distribution with two peaks. A total of 67% and 5% of patients had past history of syncope and injury due to syncope, respectively. Of the patients, 38% underwent Holter monitoring, 79 (87%) underwent head-up tilt table test (HUTT) test, 8% underwent treadmill test, 36% underwent CT/MRI of the brain, 25% underwent electroencephalography, 40% underwent carotid sinus massage, 7% underwent coronary angiography, 3% underwent electrophysiological study, and 3% of patients underwent carotid Doppler ultrasound. The commonly noted syncope was vasovagal/neutrally mediated syncope (68%). However, the etiology of syncope could not be determined in six (7%) patients.

Conclusion: This study concluded that the initial evaluation of patients with syncope should focus on history, physical examination, and ECG examination. Information obtained from such basic evaluations should be used to guide the selection of further high yield tests to reduce the cost of evaluation and for appropriate workup for the diagnosis of syncope.

## Introduction

Syncope is a common medical condition defined as a sudden, temporary loss of consciousness, associated with loss of postural tone due to transient global cerebral hypoperfusion that is characterized by rapid and spontaneous recovery. It accounts for about 1% of hospital admissions and 3% of patients visiting the emergency department. Syncope is a significant clinical problem that can lead to injury and is sometimes the only warning sign preceding sudden cardiac death, and, if survived, it markedly reduces the quality of life [1-3]. The prevalence of the first episode of syncope is particularly high between the age of 10 and 20 years. Additional peaks in the prevalence of first syncopal episodes occur at approximately 60 and 80 years of age [4,5].

Syncope represents a vast etiology and differential diagnosis. Some disorders such as vasodepressor syncope are benign problems that may have few consequences, whereas other diseases such as ventricular tachycardia are life-threatening illnesses that require prompt evaluation and treatment. Various studies and clinical experiences have shown that accurate history, physical examination, and electrocardiography (ECG) are sufficient to reach a diagnosis and to determine the etiology of syncope in many patients. However, the cause of syncope often remains undetermined even after a detailed workup and for some, it remains a diagnostic dilemma that makes future evaluation more challenging and may entail a variety of diagnostic tests, consultations, and hospitalizations [6-9].

Some studies have used different algorithms for the evaluation of patients with syncope and to minimize unnecessary testing [8-14]. Such an approach led to an increase in the percentage of patients with confirmed etiology but did not help in decreasing the cost of diagnostic workups or the use of low-yield tests. Therefore, here we studied the clinical profile of patients with syncope and the usefulness of various tests to reach a diagnosis of syncope and its etiology.

## Materials and methods

This was a cross-sectional, observational study conducted at a tertiary care center in India from April 2018 to March 2019. The study was approved by the Institutional Ethics Committee, Kasturba Medical College, Mangaluru (IEC KMC MLR 02-18/21) and was conducted as per good clinical practice and the Declaration of Helsinki. All the patients gave written informed consent before enrollment in the study.

The study included consecutive patients (aged ≥ 12 years) who presented with a complaint of syncope (inpatient or outpatient) during the study period. Individuals with near syncope and age less than 12 years were excluded from the study.

Detailed information was obtained from each enrolled patient on history, physical examination, and appropriate investigations (as needed). Pertaining to history, patients were asked regarding circumstances in which syncopal episodes occurred: emotional trigger, prolonged standing, particular posture, presence or absence of prodrome, and association with chest pain or palpitations. History was also obtained regarding previous episodes of syncope, their frequencies, age during the first episode of syncope, and occurrence of injuries due to syncopal episodes. During questioning about the history of syncope, justification from eyewitnesses was also taken into consideration.

Drug history was also obtained as medicinal drugs and drugs of illicit use could also lead to syncope. The particular emphasis being on antihypertensive drugs (β-blockers, calcium channel blockers, angiotensin receptor blockers, angiotensin-converting enzyme inhibitors), diuretics, antidepressants, antipsychotics, and antiarrhythmics. Any significant treatment history apart from drug history was also noted. Complete physical examination was carried out for all patients in the study including pulse, blood pressure with postural variation, anemia, and cyanosis along with other general examinations. Cardiovascular and other physiological examinations were also done.

All patients underwent ECG and echocardiographic examinations. ECG monitoring with Holter’s criteria was done if an arrhythmia was suspected. Head-up tilt table test (HUTT) was done when neurogenic/vasovagal syncope and orthostatic hypotension were suspected. Carotid sinus massage was carried out for patients aged more than 40 years. A treadmill test (TMT) was done if exercise-induced arrhythmia or ischemia was suspected. Electroencephalography (EEG), neuroimaging (CT/MRI brain), and carotid-vertebral arterial Doppler were done when there was a suspicion of neurological cause for the patient’s transient loss of consciousness. A coronary angiogram was done when myocardial ischemia was suspected as a cause of syncope. Other tests performed were hemoglobin, random blood sugar, and serum electrolytes (Na+ and K+). Among various tests/investigations carried out on a patient, a note was made about which test helped in identifying the etiology of the syncopal episode.

Diagnosis of syncope and its different types

Vasovagal Syncope

Also known as neurally mediated syncope, syncope is considered vasovagal syncope when it is associated with a trigger, which may include prolonged standing, orthostatic stress, or emotional stress, and typical prodrome includes lightheadedness, giddiness, blurred vision, sweating, pallor, nausea, etc.

Orthostatic Syncope

If syncope happened while the patient was in a standing posture with documentation of orthostatic hypotension, it is known as orthostatic syncope.

Situational Syncope

Syncope is considered situational syncope when a specific situation acted as the trigger for a patient’s syncopal episode such as micturition, defecation, coughing, sneezing, and swallowing.

Cardiogenic Syncope

Syncope is considered cardiogenic syncope when it resulted due to either (i) cardiac ischemia (as evidenced by ECG suggestive of acute ischemia), (ii) cardiac arrhythmia (as detected by ECG, Holter, TMT, or electrophysiological study), or (iii) cardiovascular structural disease (atrial myxoma, severe aortic stenosis, pulmonary hypertension, pulmonary embolus, or acute aortic dissection).

Statistical analysis

All data were analyzed using SPSS version 20 (IBM Corp., Armonk, NY). Continuous and categorical variables are presented as mean ± standard deviation and frequency (percentage), respectively.

## Results

A total of 90 patients (inpatients and outpatients) who presented with syncope were enrolled in the study, of which 41 (45%) were males. In this study, the age distribution of patients with syncope showed a bimodal distribution with two peaks: one in adolescents and young adults (12-20 years) consisting of 29 (32%) patients and the second in elderly (>60 years) consisting of 28 (31%) patients. A total of 61 (67%) patients had a history of syncope and four (5%) patients had a history of injury due to syncope.

Among all, 22 (48%) patients had hypertension, 10 (22%) had diabetes mellitus, and five (11%) had ischemic heart disease. In this study, ECG was performed in all enrolled patients, of which 59 (66%) showed normal ECG. The ST-T changes suggestive of ischemia were noted in six (7%) patients. In addition, 31 (35%) patients underwent Holter (ambulatory ECG) monitoring, of which 16 (52%) patients had normal Holter findings, two (7%) showed sinus bradycardia, two (7%) showed significant ventricular arrhythmia, and significant ventricular premature complexes were noted in four (13%) patients. Table 1 depicts the baseline demographic characteristics of all enrolled patients with syncope.

**Table 1:** Baseline demographic characteristics of all enrolled patients with syncope.

Characteristics	N = 90; n (%)
Age	
12-20 years	29 (32%)
21-30 years	7 (8%)
31-40 years	9 (10%)
41-50 years	7 (8%)
51-60 years	10 (11%)
>60 years	28 (31%)
Gender	
Male	41 (45%)
Female	49 (55%)
History	
History of syncope	61 (67%)
History of Injury due to syncope	4 (5%)
Comorbidities (age > 40 years)	n = 45
Hypertension	22 (48%)
Diabetes mellitus	10 (22%)
Ischemic heart disease	5 (11%)
Type of syncope	
Vasovagal/neurally mediated syncope	61 (68%)
Orthostatic hypotension	5 (6%)
Cardiogenic syncope	13 (14%)
Carotid sinus hypersensitivity	2 (2%)
Psychogenic/pseudosyncope	3 (3%)
Undetermined etiology	6 (7%)

The overall findings of important tests in all enrolled patients are outlined in Tables 2, 3.

**Table 2:** ECG pattern and Holter findings in patients with syncope.

Variables	No. of patients (n = 90) (%)
ECG pattern	
Normal	59 (66%)
Left anterior hemiblock	3 (3%)
Left ventricular hypertrophy	4 (5%)
Ventricular premature complexes	3 (3%)
Atrial premature complexes	2 (2%)
ST-T changes (ischemia)	6 (7%)
Left bundle branch block	-
Right bundle branch block	2 (2%)
Congenital heart block	1 (1%)
Atrial fibrillation	2 (2%)
Sinus bradycardia	3 (3%)
Others	5 (6%)
Holter findings (n = 31)	
Normal	16 (52%)
Sinus tachycardia	1 (3%)
Ventricular premature complexes	4 (13%)
Paroxysmal atrial fibrillation	1 (3%)
Atrial tachycardia/supraventricular tachycardia	1 (3%)
Sick sinus syndrome	3 (9%)
Significant ventricular arrhythmia	2 (7%)
Advanced atrioventricular block	1 (3%)

**Table 3:** Tilt table and coronary angiographic findings in patients with syncope.

Type of response in tilt table test-positive patients for vasovagal syncope (n = 56)	
Type 1 - mixed response	39 (69%)
Type 2 - cardioinhibitory response	5 (9%)
Type 3 - vasodepressor response	12 (22%)
Coronary angiographic findings (n = 7)	
Normal	1 (14%)
Non-obstructive coronary artery disease	1 (14%)
Single vessel disease	3 (44%)
Double vessel disease	2 (28%)
Triple vessel disease	-

Out of 90 patients, 79 underwent HUTT, which was noted to be positive in 56 patients, suggestive of vasovagal syncope. Out of 56 HUTT-positive patients, 39 (69%) had type 1 mixed response, five (9%) had type 2 cardioinhibitory response, and 12 (22%) had type 3 vasodepressor response. Coronary angiography was performed in seven (7.78%) patients with syncope as they reported ST-T changes in ECG with clinical findings suggestive of ischemia. Five patients had significant coronary artery disease, one had non-significant coronary artery disease, and one showed normal angiogram.

In our study, the most commonly noted syncope was vasovagal/neutrally mediated syncope (68%), followed by cardiogenic syncope (14%), orthostatic hypotension (6%), carotid sinus hypersensitivity (2%), and psychogenic/pseudosyncope (3%). However, the etiology of syncope could not be determined in six (7%) patients.

In this study, all patients with syncope underwent ECG and echocardiographic examination. A total of 31 (38%) patients underwent Holter, 79 (87%) underwent HUTT, seven (8%) underwent TMT, 33 (36%) underwent CT/MRI of the brain, 23 (25%) underwent EEG, 36 (40%) underwent carotid sinus massage, seven (7%) underwent coronary angiography, three (3%) underwent electrophysiological study, and three (3%) patients underwent carotid Doppler ultrasound.

Among all tests performed, ECG helped to determine the etiology of syncope in seven (8%) cases, echocardiography in three (4%) cases, Holter in five (16%) cases, HUTT in 59 (74%) cases, carotid sinus massage in two cases, and electrophysiological study in one case (Table 4).

**Table 4:** Diagnostic tests obtained in the evaluation of patients with syncope. ECG: electrocardiography; TMT: treadmill test; HUTT: head-up tilt table test; CT: computed tomography; MRI: magnetic resonance imaging; EEG: electroencephalography; CAG: coronary angiography; CSM: carotid sinus massage.

	Obtained, n (%)	Abnormal, n (%)	Helped determine etiology, n (%)
ECG	90 (100%)	31 (34%)	7 (8%)
Echocardiography	90 (100%)	20 (22%)	3 (4%)
Holter	31 (38%)	8 (26%)	5 (16%)
TMT	7 (8%)	-	-
HUTT	79 (87%)	59 (74%)	59 (74%)
CT/MRI (brain)	33 (36%)	8 (24%)	-
EEG	23 (25%)	-	-
Carotid Doppler ultrasound	3 (3%)	-	-
CAG	7 (7%)	5 (71%)	-
CSM	36 (40%)	2 (5%)	2 (5%)
Electrophysiological study	3 (3%)	1 (33%)	1 (33%)

CT/MRI of the brain, EEG, carotid Doppler ultrasound, TMT, and coronary angiography did not help in determining the etiology of syncope.

## Discussion

We carried out a cross-sectional, observational study on the clinical profile of patients with syncope. Diagnosis and etiology of syncope in most patients get established with accurate history and physical examination. Various tests and investigations, when guided by initial evaluation, help to confirm the etiology as well as are useful for patients with unexplained syncope at initial evaluation. We also studied the utility of various diagnostic tests in the evaluation of patients with syncope.

Among the enrolled patients with syncope, age distribution showed a bimodal peak. One peak was in the adolescents and young adult group, and the second peak was seen in the elderly group (>60 years), which was consistent with prior studies. In addition, a total of 45 patients with syncope were above 40 years of age. The presence of comorbid conditions like hypertension, diabetes mellitus, ischemic heart disease, and drug therapy for these conditions can contribute to the etiopathogenesis of syncope in these patients. Syncope is often a recurring condition. A significant number of patients with syncope also have a history of similar complaints and syncope, which can interfere with the quality of life and can sometimes be life-threatening. In our study, 67% of patients had a history of syncope and 5% of patients had sustained injury due to syncope. Various studies have shown an association between cardiovascular diseases, medications for such diseases, occurrence/recurrence of syncope, and even sudden cardiac death. Association between syncope and cardiovascular diseases and medications is helpful for risk stratification in the emergency department as well as evaluation/management of patients with syncope.

Generally, ECG is obtained in all patients who present with syncope, but studies have shown that ECG is not considerably useful to establish the diagnosis and etiology of syncope. However, ECG is very helpful to exclude serious etiologies such as cardiac arrhythmias at the initial stages. In our study, all patients underwent ECG examination, which was normal in 66% of patients. ECG helped in determining the etiology of syncope in 8% of patients, which was in line with previous findings [15,16]. Furthermore, continuous ECG monitoring is indicated when there is a high likelihood of arrhythmia as an etiology of syncope. In clinical practice, Holter monitoring (ambulatory ECG monitoring) is done for 24-72 hours. In our study, Holter monitoring helped in determining etiology in five (16%) patients and these patients showed significant ventricular arrhythmia and sick sinus syndrome on Holter.

The study also obtained echocardiograms for all patients, which was abnormal in 22% of patients and helped determine etiology in three (4%) patients, of which two had severe valvular aortic stenosis and one had a pulmonary embolism. Similarly, previous studies also stated that echocardiography was used in the diagnosis of etiology of syncope in 2-3% of patients [14,15,17-19]. TMT is recommended for the evaluation of patients who had syncope during or immediately after exertion, as it is useful for the evaluation of ischemic heart disease when cardiac arrhythmia, particularly ventricular arrhythmia, is suspected as a cause of syncope. However, previous studies had shown that yield of TMT in the diagnosis of the etiology of syncope is very low (<1%) and even in the present study, TMT did not help in determining the etiology of syncope, as among 8% of patients who underwent TMT, all turned out to be normal.

In our study, 79 (87%) of 90 patients underwent HUTT on the assumption of neurally mediated or vasovagal syncope from history and physical examination. In 70% of patients, HUTT confirmed the diagnosis of vasovagal syncope, and in 4% of patients, it led to the diagnosis of orthostatic hypotension, and thus the diagnostic yield of HUTT in our study was 74%. In a study performed by Mitro et al. [17], the diagnostic yield of the tilt table test was found to be 52%. Similarly, another study by Prakash et al. [20] reported a 64% diagnostic yield of the tilt table test. Furthermore, in the present study, out of 56 patients who showed a positive tilt table test for vasovagal syncope, 22% had a vasodepressor response, 9% had a cardioinhibitory response, and 69% had a mixed response.

Carotid sinus massage is indicated when symptoms suggest a possibility of carotid sinus syncope (carotid sinus hypersensitivity) and in cases of unexplained syncope in elderly patients. In our study, we carried out carotid sinus massage in 40% of patients with age > 40 years. The massage was done in both supine and standing positions (during the tilt table test). Two patients were found to have carotid sinus hypersensitivity, thus the diagnostic yield of carotid sinus massage was 5%, which was in line with already reported findings [16,17].

Seizure and cerebrovascular accidents, mainly transient ischemic attacks, are the important differential diagnosis for patients who are suspected of having syncopal episodes. In the majority of cases, differentiation can be achieved through accurate history. Still, it is seen that many of such patients undergo neurological tests such as EEG, MRI, or CT scan of the brain and carotid Doppler ultrasound. The present study, in support of other previous similar studies, has shown that these tests have a very low diagnostic yield for syncope [17,21-24].

In this study, three patients underwent electrophysiological examination, of which two had unexplained syncope, with ECG suggestive of possible arrhythmic etiology, but the study turned out to be normal. One adolescent patient showed congestive heart block on ECG, so she underwent electrophysiological study and was found to have primary conduction abnormality. In our study, very few patients underwent electrophysiological study, so diagnostic yield cannot be determined. On the contrary, previous studies reported a very high diagnostic yield of syncope with electrophysiological studies [17]. In our study, seven patients underwent coronary angiography; however, it did not help in determining the etiology of syncope.

Thus, the key findings of the study are as follows: (i) ECG and echocardiography were useful in excluding serious etiologies rather than finding out the etiology; (ii) Holter monitoring was helpful for patients who have frequent syncopal episodes and arrhythmogenic etiology was suspected; and (iii) carotid sinus massage was useful for evaluation of unexplained syncope in elderly and when carotid sinus hypersensitivity is suspected.

Limitations

The limitations of this study include its observational, single-center nature with a smaller number of enrolled patients. Moreover, factors that might have affected the attending physician's decision on the order of some tests were not considered in this study. Thus, larger studies on heterogeneous patient populations are required to validate this evidence.

## Conclusions

The findings of the present study concluded that the initial evaluation of patients with syncope should focus on history, physical examination, and ECG examination. However, ECG is not reliable in some cases as resting ECG fails to detect myocardial ischemia. Therefore, information obtained from such basic evaluations should be used to guide the selection of further high yield tests.

Such an approach can provide a cost-effective way for the evaluation and workup of patients with syncope. Moreover, larger studies are required to validate the current findings.
